# A minimalist model to measure interactions between proteins and synaptic vesicles

**DOI:** 10.1038/s41598-020-77887-1

**Published:** 2020-12-03

**Authors:** Eleonora Perego, Sofiia Reshetniak, Charlotta Lorenz, Christian Hoffmann, Dragomir Milovanović, Silvio O. Rizzoli, Sarah Köster

**Affiliations:** 1 Institute for X-Ray Physics, University of Göttingen, 37077 Göttingen, Germany; 2 Institute for Neuro- and Sensory Physiology, Center for Biostructural Imaging of Neurodegeneration, University Medical Center Göttingen, 37075 Göttingen, Germany; 3 Laboratory of Molecular Neuroscience, German Center for Neurodegenerative Diseases (DZNE), 10117 Berlin, Germany; 4 Cluster of Excellence “Multiscale Bioimaging: from Molecular Machines to Networks of Excitable Cells” (MBExC), University of Göttingen, 37073 Göttingen, Germany

**Keywords:** Single-molecule biophysics, Biophysics, Vesicle trafficking, Neuroscience, Molecular neuroscience, Biochemistry

## Abstract

Protein dynamics in the synaptic bouton are still not well understood, despite many quantitative studies of synaptic structure and function. The complexity of the synaptic environment makes investigations of presynaptic protein mobility challenging. Here, we present an in vitro approach to create a minimalist model of the synaptic environment by patterning synaptic vesicles (SVs) on glass coverslips. We employed fluorescence correlation spectroscopy (FCS) to measure the mobility of monomeric enhanced green fluorescent protein (mEGFP)-tagged proteins in the presence of the vesicle patterns. We observed that the mobility of all eleven measured proteins is strongly reduced in the presence of the SVs, suggesting that they all bind to the SVs. The mobility observed in these conditions is within the range of corresponding measurements in synapses of living cells. Overall, our simple, but robust, approach should enable numerous future studies of organelle-protein interactions in general.

## Introduction

Information processing in the brain depends on the transmission of information at the level of inter-neuronal synapses. The large majority of the brain synapses base their activity on the release of neurotransmitters from the presynaptic side (the so-called synaptic bouton) onto receptors on the plasma membrane of the postsynaptic cells. This process takes place through the fusion of neurotransmitter-filled synaptic vesicles (SVs) with the presynaptic plasma membrane (exocytosis), followed by the retrieval of SV proteins from the membrane (endocytosis), and the formation of new SVs, which are ready for function^1,2^. The composition of the synaptic bouton reflects the importance of neurotransmitter release, as much of its space is taken up by the SVs, organized in a dense cluster^3^. Exo- and endocytosis cofactor proteins are also prominent in the bouton, where they tend to be strongly enriched^4^.

Synaptic transmission has been the subject of many quantitative studies, which have established, for example, the copy numbers of many of the vesicular^5^ or presynaptic proteins^4^. The spatial distribution of the proteins has also been analyzed^4^, and recently the average mobility of multiple synaptic proteins has been estimated^6^. However, in spite of the rich quantitative information available on synaptic transmission, the true molecular organization of the presynaptic bouton is still unclear. Importantly, it is still unresolved, why the soluble synaptic proteins are enriched in the bouton. They must be retained locally by mechanisms that apply to most or all of the exo- and endocytosis cofactors, as otherwise they would be lost in the axon, which can be many orders of magnitude larger in volume than the synapse^7^. The nature of these mechanisms is not yet understood, despite more than a decade of research on this subject. As many presynaptic proteins colocalize well with the SVs, it has been proposed that the vesicles bind them, thereby serving as a form of storage container for such proteins^7,8^. This concept has more recently been extended to include the hypothesis that the clustered SVs form a distinct liquid phase, together with some of the presynaptic proteins^9,10^. While this hypothesis is highly attractive, a formal demonstration of its main assumption, namely that multiple exo- and endocytosis cofactors interact strongly with the SVs, is still missing. Biochemical experiments have suggested that isolated SVs can collect soluble proteins on their surfaces^5,7^, but the interpretation of such experiments is difficult, since most of the tested proteins failed to enrich on purified SVs^5^.

To test the interaction of presynaptic proteins with the SVs in a more direct and controlled fashion, it is necessary to analyze the behavior of individual proteins in a highly defined system, in which both the vesicles and the proteins can be introduced in standardized conditions, and in the absence of most cellular components. Here, we present a minimalist in vitro model of the synaptic environment that enables such controlled measurements. Using a micro-patterning strategy, SVs were immobilized on glass coverslips, creating a 2D vesicle cluster, where protein dynamics could be investigated in a straightforward manner. We employed fluorescence correlation spectroscopy (FCS) to quantify the mobility of eleven different synaptic proteins in this system, and we found a dramatic decrease of the protein mobility in the presence of the SVs, confirming an effective interaction between each of the proteins and the SVs. Importantly, we found a strong agreement of the in vitro diffusion coefficients and corresponding values measured in living cells^6,11–13^. This result implies not only that such proteins interact with the vesicles, but also that their mobility is governed by this interaction, confirming the hypothesis that the SV cluster is a major factor in the organization of presynaptic proteins^7–9^.

## Results and discussion

### An in vitro replica of the synapse

**Figure 1:**
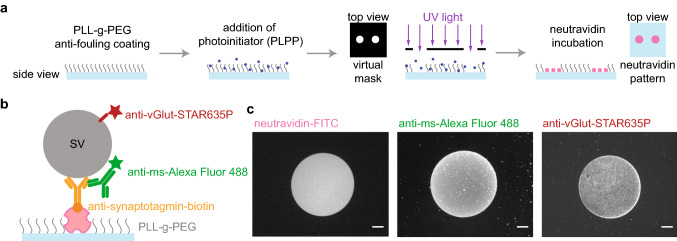
Patterning strategy and SV immobilization. (**a**) Schematic representation of photopatterning of neutravidin on a glass coverslip. The glass coverslip was uniformly coated with an anti-fouling layer of PLL-g-PEG. The photoinitiator (PLPP) was added on top of the PLL-g-PEG layer and the substrate was exposed to UV light through a virtual photomask to activate the PLPP. Under UV light, the PLPP degraded the PLL-PEG layer, leaving accessible regions for neutravidin to attach. (**b**) Schematic of our strategy to attach the SVs to the substrate. The purified SVs (gray) were attached to the neutravidin (pink) functionalized glass coverslips via a biotinylated mouse anti-synaptotagmin antibody (orange). The attachment of antibodies was controlled by a secondary anti-mouse antibody labeled with Alexa Fluor 488 (green). To image the SVs, a single-domain antibody against vGLUT1, labeled with STAR635P (red) was employed. (**c**) Fluorescence micrographs of the individual steps of the vesicle immobilization strategy. The neutravidin functionalization step was assessed by fluorescently labeled neutravidin (neutravidin-FITC, left), the attachment of the biotinylated mouse anti-synaptotagmin antibody was tested with a secondary anti-mouse antibody labeled with Alexa Fluor 488 (center), and the SV attachment was tested with a single-domain antibody against vGLUT1 labeled with STAR635P (right). Note that the uniform fluorescence in the right image shows the bound SVs, whereas the small bright spots are aggregates. The images of neutravidin-FITC and anti-ms-Alexa488 were taken on two different glass coverslips. In the actual experiments, we used unlabeled neutravidin, so as to not interfere with the fluorescence of the mEGFP-tagged proteins. The scale bars are 25 μm.

Measuring protein diffusion and interaction directly in the synapse is challenging, owing to the complex and crowded environment^4^. Therefore, we designed a minimalist in vitro version of the synapse, composed of two-dimensional SV arrays adhered to a glass surface, with which we studied protein mobility in a highly controlled manner. To attach SVs to glass coverslips, we first functionalized the substrates with patterns of neutravidin. The size and the shape of the patterns are of little relevance for the present study, albeit the method can be adapted to many other research questions, where these parameters may play a role. In the present case, we chose to pattern the surface instead of uniformly coating it, because it provides a straightforward way of controlling the success of the subsequent steps by comparing the pattern to the uncoated regions nearby. The process, as well as the quality control, is shown in Fig. 1. A passivated glass coverslip was exposed to UV light through a virtual photomask, which projects the pattern onto the substrate employing digital micromirror devices (DMD), thus avoiding the use of a real mask. For more details on this procedure, see Ref. 14. The photo-initiator (4-benzoylbenzyl-trimethylammonium chloride, sold as PLPP (Product of Liaison for Protein Patterning) by Alvéole, Paris, France) degraded the anti-fouling layer of poly(L-lysine)-graft-poly(ethylene glycol) (PLL-PEG) upon exposure, making the exposed area accessible to neutravidin (pink in Fig. 1a,b). The reliable attachment of neutravidin was tested using a fluorescently labeled variant, see Fig. 1c, left. The SVs (gray in Fig. 1b) were attached via a biotinylated anti-synaptotagmin antibody (orange in Fig. 1b). Synaptotagmin is an abundant vesicle protein, which is essential for both exo- and endocytosis, and which is presumably present in all functional vesicles, thereby rendering it a convenient tool for anchoring the SVs to the neutravidin. The proper attachment of the biotinylated anti-synaptotagmin antibodies was tested via a secondary anti-mouse-Alexa Fluor 488 antibody (green in Fig. 1b). As shown in Fig. 1c, center, the antibody attachment is well defined and with a low background. To check for unspecific interactions to the patterned neutravidin, a secondary antibody (anti-ms-STAR635) was incubated on the neutravidin pattern without the biotinylated anti-synaptotagmin antibody added. As shown in Fig. S1a, no signal was obtained from the secondary antibody. Finally, to assess the attached SVs, a single-domain antibody against vGLUT1 labeled with STAR635P was employed (red in Fig. 1b). vGLUT1 is a major neurotransmitter transporter and can therefore be used to detect the vesicles. As shown in Fig. 1c, right, the signal stemming form the vesicle pattern was well confined to the circle region and displayed low background. As a negative control, the single domain antibody against vGLUT1 labeled with STAR635P was incubated on functionalized coverslips without SVs. As shown in Fig. S1b, no unspecific interaction was found.

**Figure 2:**
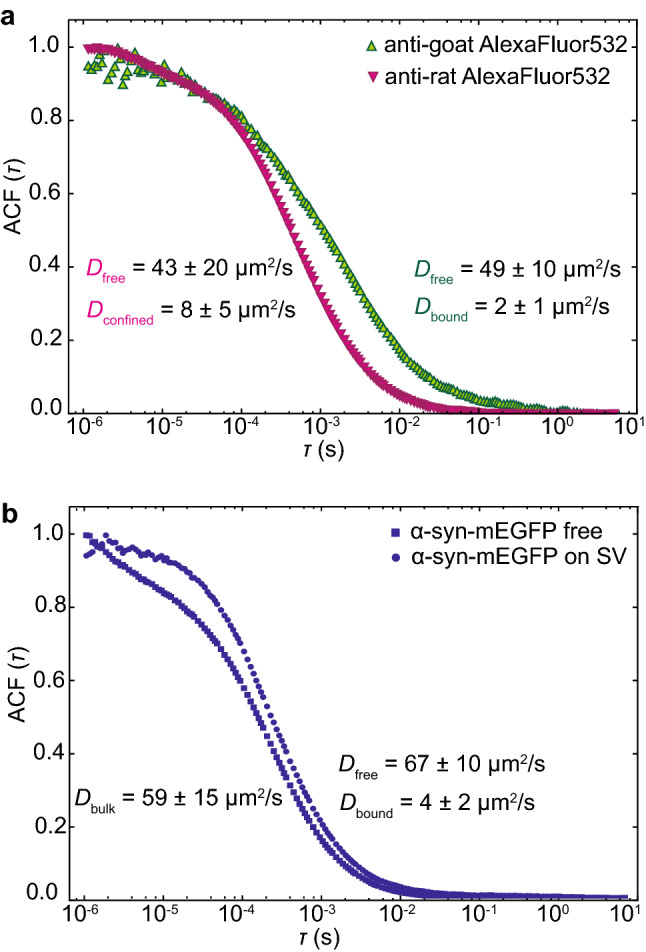
Control measurements for the newly developed assay. (**a**) ACF curves (averaged data from 25 single curves) of anti-goat-Alexa Fluor 532 (green upright triangles) and anti-rat-Alexa Fluor 532 (magenta inverted triangles) in the presence of SV patterns. For both antibodies, two diffusion coefficients were retrieved from the fits. The values are reported in the graph (color coded). The anti-goat-Alexa Fluor 532 interacted with the patterned SVs; the diffusion coefficient $D_{\text {bound}}$ corresponds to interacting antibodies and $D_{\text {free}}$ to non-interacting, i.e. freely diffusing antibodies. Although anti-rat-Alexa Fluor 532 is not expected to interact with the vesicles, we observed two diffusion coefficients; $D_{\text {free}}$ corresponds to freely diffusing antibodies, whereas $D_{\text {confined}}$, corresponds to a slower component, which we attribute to the crowded environment in the vesicle layer. (**b**) ACF curves (averaged data from 20 single curves) of α-synuclein-mEGFP in the presence of SVs (blue squares) and for comparison without the vesicle pattern (blue circles). As expected, without the vesicle pattern, we obtained the bulk diffusion coefficient of α-synuclein-mEGFP, $D_{\text {bulk}}$; by contrast, in presence of the SV pattern, we observed two diffusion coefficients; $D_{\text {free}}$ is very similar to the bulk diffusion coefficient, $D_{\text {bound}}$ is much smaller and corresponds to α-synuclein-mEGFP interacting with the SVs.

To verify our approach of measuring protein mobility in the presence of a 2D SV array, we performed two control experiments. We targeted the abundant vesicular glycoprotein synaptophysin with primary rabbit anti-synaptophysin antibodies and secondary goat anti-rabbit antibodies, and thereby visualized the vesicles. The first control was provided by the use of diffusing anti-goat-Alexa Fluor 532 antibodies, which interact with the antibody complex on the SVs, whereas as a second control we used diffusing anti-rat-Alexa Fluor 532 antibodies, which are not expected to bind to the system. Figure 2a shows the average normalized autocorrelation function (ACF) (average data from 25 ACFs each) for both control antibodies, measured in the presence of SVs. We focused on the surface of our substrate with the SVs attached and thus ensured that the measured diffusion took place in the crowded environment of the layer of SVs to which the proteins and antibodies can bind. For the interacting antibody, anti-goat-Alexa Fluor 532 (green upright triangles in Fig. 2a), two diffusion coefficients were retrieved from fitting the data. The first diffusion coefficient, $D_{\text {free}}$, reflects the non-interacting component, i.e. the freely diffusing antibodies, with a value of (49 ± 10) μm^2^/s. This value is similar to other measurements of freely diffusing antibodies^15^ and to a value of $D_{\text {bulk}}$ = (55 ± 20) μm^2^/s which we obtained for the same antibody measured in bulk, i.e. without the SV pattern. The second diffusion coefficient $D_{\text {bound}}$ is much smaller, (2 ± 1) μm^2^/s, and we interpret it as stemming from antibodies interacting with the SVs. Note that the two separate “ensembles” of freely diffusing and interacting, or bound, antibodies, respectively, correspond to average amounts of time for each antibody spent in “bound” and in “free” states. Interestingly, although anti-rat antibodies are not supposed to bind to anything in the SV array, two diffusion coefficients were also retrieved from the analysis of the corresponding data (magenta inverted triangles in Fig. 2a). Again, we observed one diffusion coefficient of $D_{\text {free}}$ = (43 ± 20) μm^2^/s that we attribute to the freely diffusing component as verified by a bulk measurement without an SV pattern ($D_{\text {bulk}}$ = (47 ± 29) μm^2^/s). A more slowly diffusing component was additionally measured, with $D_{\text {confined}}$ = (8 ± 5) μm^2^/s. We speculate that this component describes confinement effects caused by the dense 2D vesicle pattern. Such confinement may slow down the mobility of the antibody^16–18^.

As an additional test for our newly developed assay, we compared the diffusion of purified α-synuclein-mEGFP (monomeric enhanced green fluorescent protein) with and without the SV pattern, as shown in Fig. 2b. α-synuclein is a SV-binding protein particularly important in Parkinson’s disease. When α-synuclein-mEGFP was measured in bulk, without the SV pattern present (blue circles in Fig. 2b), a diffusion coefficient of $D_{\text {bulk}}$ = (59 ± 15) μm^2^/s was obtained. This diffusion coefficient is smaller than the ones found in literature^19,20^ and by us (for α-synuclein-Alexa Fluor 532, *D* = (102 ± 20) μm^2^/s) when the protein is labeled with chemical dyes.

We attribute this difference to the heavy fluorescent tag (mEGFP) employed here. When α-synuclein-mEGFP was measured in the presence of the SVs, two diffusing components were retrieved from the analysis of the ACF (blue squares in Fig. 2b). The first diffusion coefficient, $D_{\text {free}}$, again corresponds to the non-interacting component, i.e. freely diffusing protein, with a value of (67 ± 10) μm^2^/s. The second diffusion coefficient, $D_2$, is much smaller, $D_{\text {bound}}$ = (4 ± 2) μm^2^/s, which we attribute to α-synuclein interacting with the SVs.

### Mobility and binding efficiency of synaptic proteins

**Figure 3:**
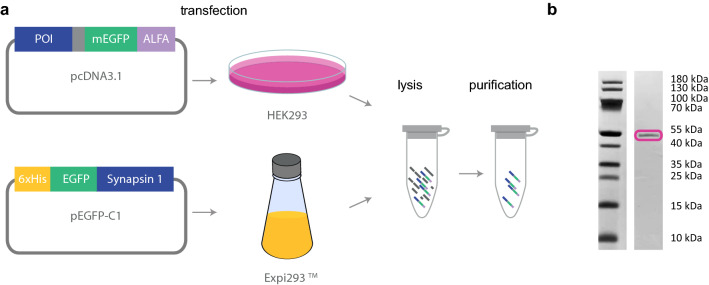
Protein purification strategy. (**a**) The protein of interest (POI, blue) fused to mEGFP (green) and ALFA-tag (purple) or His tag (orange) in the case of synapsin was expressed in HEK293 or Expi293 cells, which were then lysed. The protein was then purified using an ALFA-tag purification system^21^ or Ni-NTA purification with consecutive size exclusion chromatography. (**b**) Example of a coomassie stained polyacrylamide gel showing, as an example, purified α-synuclein. The magenta box indicates the main band, which runs at the expected molecular weight. The full-length coomassie stained polyacrylamide gel for the remaining proteins is shown in Supplementary Fig. S2.

To test the interaction of SVs and different proteins, we relied on several synaptic proteins, which are known to be involved in synaptic transmission. All synaptic proteins considered here were expressed in HEK293 cells, except synapsin and EGFP, which were expressed in Expi293 cells. The cells were lysed and the lysate was either used as is or further purified. For purification, an ALFA-tag system^21^ was employed, as described in the Materials and Methods section. Synapsin and EGFP were purified via NiNTA columns and size exclusion chromatography. A schematic representation of the protein production and purification process is shown in Fig. 3a, and the resulting purified fraction of α-synuclein is shown in Fig. 3b. The coomassie stained polyacrylamide gel for the remaining proteins is shown in Supplementary Fig. S2.

**Figure 4:**
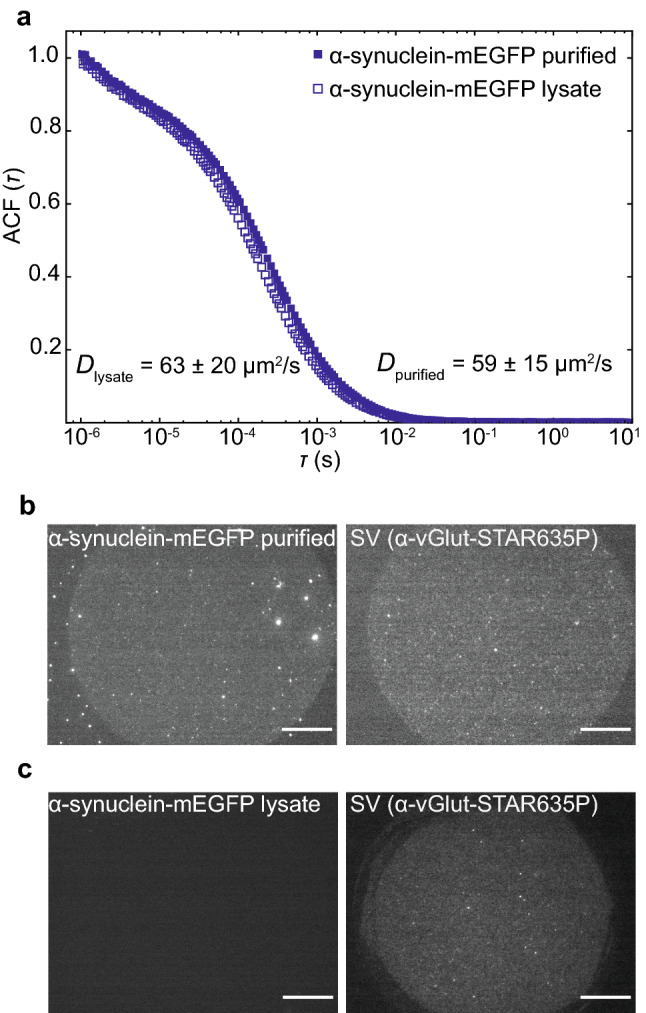
Comparison of cell lysate and purified α-synuclein-mEGFP. (**a**) Average normalized ACF of purified α-synuclein-mEGFP (closed squares, *N* = 30) and α-synuclein-mEGFP from HEK cell lysate (open squares, *N* = 30); there was no SV pattern present in these experiments; both protein preparations show the same diffusion behavior. (**b**) The purified α-synuclein-mEGFP interacted with the SV pattern (left hand side), as shown by the fluorescence signal (right; SV pattern visualized by anti-vGlut-STAR635P). (**c**) By contrast, α-synuclein-mEGFP from the cell lysate (left) did not bind to the SV pattern (right). The images in (**b**) and (**c**) were recorded after 2 hours of incubation of the SV layer with α-synuclein-mEGFP. The scale bars are 25 μm. Note that the uniform fluorescence in the right images in (**b**) and (**c**) shows the bound SVs, whereas the small bright spots are aggregates.

We characterized the diffusion properties of cell lysate containing α-synuclein-mEGFP by FCS (see Fig. 4a, open blue squares), and compared it to α-synuclein-mEGFP purified from cell lysate (closed blue squares). The ACFs as well as the diffusion coefficients derived from the fits, (63 ± 20) μm^2^/s and (59 ± 15) μm^2^/s, respectively, are in very good agreement, suggesting that the mobility of α-synuclein-mEGFP is identical before and after purification. In a next step, we investigated the ability of both versions of α-synuclein-mEGFP to interact with a SV pattern. We incubated the preparation in the presence of the SV pattern for two hours, and took fluorescence images of the mEGFP signal. For the purified α-synuclein, a fluorescent pattern (see Fig. 4b, left) similar to the SV signal (right) was visible in the GFP channel, indicating binding of the protein to the SVs. Using the non-purified cell lysate, as shown in Fig. 4c, the fluorescent pattern was not detectable in the GFP channel (left), although the SV pattern was present (right), indicating a reduced or null binding. We speculate that additional components in the cell lysate unspecifically bind to the SVs, thus blocking the binding sites for the proteins under investigation. Based on these results, we performed all remaining experiments with purified proteins, which simplified the assay, enabling us to concentrate solely on the interaction of one protein with the SVs, without the conflicting presence of all other proteins from the lysate.

### Synaptic protein mobility in the presence of SVs

After evaluating our in vitro model of a synapse using purified α-synuclein-mEGFP, we investigated ten additional soluble proteins. All these soluble proteins are known to be present in the synaptic bouton, and are important for different synaptic processes^1,2,22^. In brief, Rab3 is thought to be involved in the so-called priming process, preparing vesicles for exocytosis. Complexin 1 and α-synuclein are involved in the late stages of exocytosis by interacting with the molecules fusing the vesicle to the plasma membrane (SNAREs). Clathrin, epsin, endophilin, amphiphysin and clathrin assembly lymphoid myeloid leukemia (CALM) are involved in endocytosis, from initializing membrane curvature to coating the forming vesicle. Rab7 is involved in SV sorting after endocytosis, whereas calmodulin acts as a general regulator of calcium signaling, thereby affecting both exo- and endocytosis. Finally, we also included in this analysis synapsin I, a neuronal phosphoprotein that interacts with SVs and the actin cytoskeleton, and has recently been shown to form a liquid phase with lipid vesicles^10^.

**Figure 5:**
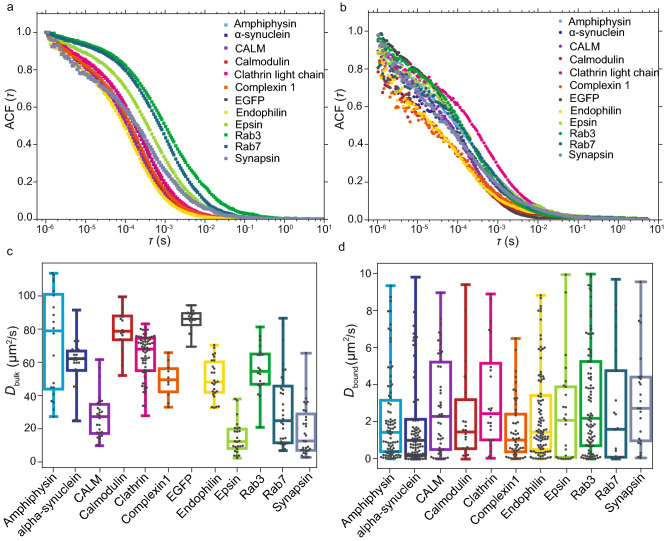
FCS measurements of all purified synaptic proteins considered here. (**a**) Average normalized FCS curves of synaptic proteins diffusing in bulk, i.e. in the absence of SVs; see legend for color code. (**b**) Average normalized FCS curves of synaptic proteins diffusing in the presence of the SVs. (**c**) Diffusion coefficients $D_{\text {bulk}}$ obtained from the fit of the FCS functions in (**a**). These diffusion coefficients reflect the 3D mobility of the proteins tagged with mEGFP in bulk. (**d**) Diffusion coefficients $D_{\text {bound}}$ obtained from fitting the FCS functions in (**b**) (excluding EGFP which did not show a bound diffusion coefficient). In this case, two diffusion coefficients were found and we show only the bound component here. Supplementary Fig. S3 shows the free component in comparison to the bulk diffusion without SVs. In (**c**) and (**d**) the boxes extend from the lower quartile to the upper quartile of the data, the middle line in each box plot represents the median, the whiskers extend from the minimum to the maximum data point. The data were plotted using Python (Python Software Foundation, Python Language Reference, version 3.7 Available at http://www.python.org).

Initially, the proteins were measured in bulk, i.e. without the SVs, to quantify the free diffusion. The averages of the ACFs for each protein (*N* varies between 18 and 70 single ACFs, 30 s of acquisition each) are reported in Fig. 5a, where each ACF was normalized to the maximum value, see legend for the color code. The distributions of the diffusion coefficients $D_{\text {bulk}}$ retrieved from the single ACF curves are shown in Fig. 5c, and Table 1 summarizes the average diffusion coefficients.

**Table 1:** Summary of the diffusion coefficients measured in the absence (bulk) and presence (bound) of SVs.

Protein	$D_\text {bulk}$ ± SD (μm^2^/s)	$D_\text {bound}$ ± SD (μm^2^/s)	% bound ± SD	$M_W$ (kDa)
Amphiphysin-mEGFP	72 ± 31	2.2 ± 2.4	24 ± 13	105
α-Synuclein-mEGFP	60 ± 16	1.8 ± 2.0	15 ± 12	44
CALM-mEGFP	28 ± 13	2.8 ± 2.5	10 ± 10	99
Calmodulin1-mEGFP	79 ± 13	2.3 ± 2.5	10 ± 11	47
Clathrin-LC-B-mEGFP	64 ± 13	3.3 ± 2.7	22 ± 18	55
Complexin1-mEGFP	50 ± 11	1.6 ± 1.6	17 ± 14	45
EGFP	85 ± 6	–	–	28
EndophilinA1-mEGFP	50 ± 12	2.4 ± 2.3	14 ± 14	70
Epsin-mEGFP	15 ± 10	2.4 ± 2.7	16 ± 11	90
Rab3a-mEGFP	55 ± 14	3.2 ± 2.9	15 ± 12	55
Rab7a-mEGFP	32 ± 23	2.8 ± 3.1	17 ± 14	53
Synapsin-EGFP	33 ± 3	3.3 ± 2.6	30 ± 18	102

The % bound shown in the fourth column corresponds to the percentage of protein that interacts at any given moment, or the time-share the protein spends in bound state. As EGFP does not interact with the vesicles, the percentage of protein bound to the SVs is zero. Values are mean ± standard deviation of at least 12 different SV patterns per protein. The last column shows the molecular weight of the respective mEGFP-fusion protein.

The large and heavy fluorescent EGFP-tag (27 kDa) decreases the diffusion coefficient of all proteins compared to the unlabeled case or the use of chemical labels, as the diffusion coefficient is proportional to the molecular weight of the diffusing molecule^23^. Relatively, this effect is more pronounced for the lighter proteins, such as α-synuclein (14 kDa) or calmodulin (16 kDa)^11^. In the case of calmodulin-mEGFP we obtained an average diffusion coefficient of $D_{\text {bulk}}$ = (79 ± 13) μm^2^/s, and for α-synuclein we obtained $D_{\text {bulk}}$ = (60 ± 16) μm^2^/s, which both are only slightly lower than the diffusion coefficient for EGFP measured to be *D* = (85 ± 6) μm^2^/s. The rigid barrel-like shape of mEGFP might indeed influence and dominate the mobility of the mEGFP-fusion protein in the case of lighter proteins^11^. However, for the larger proteins, such as epsin (63 kDa), CALM (72 kDa) and synapsin (74 kDa), a clear difference between the protein diffusion coefficients and the EGFP diffusion coefficient was observed. For endophilin A1 (43 kDa), which has a size in-between the smallest and the largest examples considered here, the measured bulk diffusion coefficient was, as expected, smaller compared to the theoretical value for the protein without mEGFP, *D* = 97 μm^2^/s, calculated using the Stokes-Einstein law^23^ and considering a radius of gyration of 48 Å^24^, and comparable, within one standard deviation, to fluorescence recovery after photobleaching (FRAP) measurements on EGFP-fused endophilin A1^25^.

When measured in the presence of SVs (Fig. 5b), the average ACFs (*N* varies between 20 and 90 single ACFs, 30 s acquisition each) were more noisy for small $\tau$, i.e. fast time scales, as shown in Figure 5b. The interaction with the SVs may lead to variations in the protein concentration in the observation volume, affecting the low-$\tau$ ACFs. Furthermore, as these measurements took place very close to the glass surface, artefacts like scattered photons or bleaching of the dye may be more pronounced^26^. In this case, we analyzed the ACFs using a two-component FCS model [Eq. (3)]. For all proteins, apart from EGFP, we found one diffusion coefficient, $D_{\text {free}}$, in the same range as the bulk values, and a second, much lower one, $D_{\text {bound}}$. For a comparison of the bulk diffusion coefficients $D_{\text {bulk}}$ and $D_{\text {free}}$, see Supplementary Fig. S3. The ACFs of EGFP can be fully described with one component only, confirming that EGFP does not interact with the SVs. In fact, the average diffusion coefficient measured on the SV patterns, *D* = (81 ± 30) μm^2^/s is comparable to the value measured in bulk. For all proteins $D_{\text {bulk}}$ and $D_{\text {free}}$ are very similar, however, in some cases $D_{\text {free}}$ is decreased to some extent in comparison to the bulk diffusion. We can only speculate about the reason for this behavior. Possibly the diffusing proteins are binding interaction partners from the vesicle surfaces, making them slightly heavier, or their mobility is decreased because of the confinement effects caused by the 2D vesicle pattern. By contrast, we attribute $D_{\text {bound}}$ to the bound component. The corresponding distributions of the diffusion coefficients $D_{\text {bound}}$ are shown as box plots in Fig. 5d, and in Table 1, the average values are reported.

**Figure 6:**
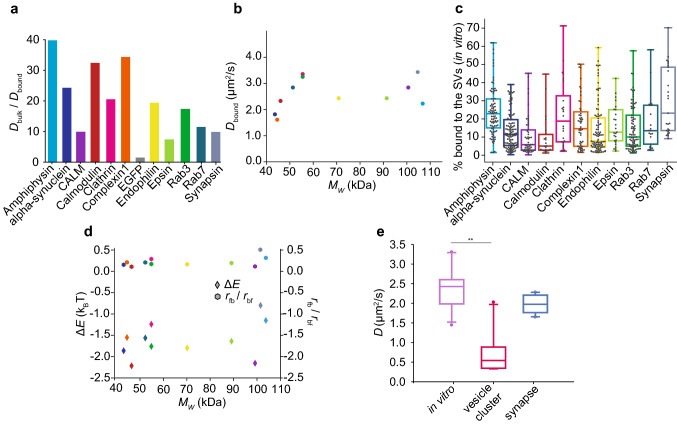
Interpretation of the diffusion coefficients measured in the presence of SVs. (**a**) Ratio between the diffusion coefficients for the non-interacting component $D_\text {bulk}$ and for the interacting component $D_\text {bound}$ for all synaptic proteins considered here. The protein mobility was considerably slowed down by interaction with the vesicles. (**b**) The average diffusion coefficients $D_{\text {bound}}$ of the bound fraction do not correlate with the molecular weights of the mEGFP-fusion proteins. The colors represent the proteins, color coded as in (**a**). (**c**) Percentage of the time that each protein remains bound. (**d**) Energy difference between the free and the bound state (left hand side axis, diamonds) measured in units of $k_B T$ and ratio between the transition rate from free to bound state, $r_{fb}$, and transition rate from bound to free state, $r_{bf}$ (right hand side axis, circles). The colors represent the proteins, color coded as in (**a**) and (**c**). (**e**) The median diffusion coefficients $D_{\text {bound}}$ of the interacting components (purple) are similar to the values obtained in synapses in living cells (blue), and are considerably higher than those obtained in the vesicle cluster in living cells (magenta)^6^. We performed a Kruskal-Wallis test followed by Tukey’s post-hoc test, and obtained *p* = 0.003 (significance level 0.05), confirming the statistical difference between $D_{\text {bound}}$ and the values obtained in vesicle clusters, and no significant difference for $D_{\text {bound}}$ and the values obtained in the synapse (*p* = 0.08). In (**c**) and (**e**) the boxes extend from the lower quartile to the upper quartile of the data, the middle line in each box plot represents the median, the whiskers extend from the minimum to the maximum data point. The data were plotted using Python (Python Software Foundation, Python Language Reference, version 3.7 Available at http://www.python.org).

The diffusion coefficients for the bound component are statistically different (Mann–Whitney test, *p*-values are presented in Table S1) from the diffusion coefficients measured without the SVs, indicating a clearly different mobility in the two cases. The decrease in mobility of synaptic proteins in the presence of SVs, between 7-fold (epsin) and 40-fold (amphiphysin), is shown by the ratio between the bulk diffusion coefficient $D_{\text {bulk}}$ and the bound diffusion coefficient $D_{\text {bound}}$, see Fig. 6a. This decrease in mobility differs between different proteins. It reflects the diffusion coefficient $D_{\text {free}}$ of the proteins, since $D_{\text {bound}}$ is very similar in all cases. In particular, the decrease in mobility is less pronounced for the heaviest proteins, CALM, epsin and synapsin, which naturally already show a lower mobility without the SVs. Interestingly, the average diffusion coefficients of the bound component $D_{\text {bound}}$ for the measured proteins do not depend on the molecular weight of the mEGFP-fusion proteins, as shown in Fig. 6b, but are all found to be on the order of 2 μm^2^/s. These results show that the decreased mobility in the presence of SVs is a property of binding and confinement effects, rather than a property of protein size. All measured proteins do, on average, interact with the SVs for at least 10 % of the time, as shown in Table 1, where the average percentages of the bound component are reported for all proteins.

We calculated the energy difference between the free and the bound state, taking advantage of Boltzmann statistics. The percentage of the bound component, shown in Fig. 6c, reflects the probability, or time-share, $p_b$ that a protein interacts with the SVs. Since we observe $p_b < 0.5$ for all proteins, the interacting state has an average higher energy than the free state. In fact, the average energy difference $\Delta E$ derived from the average probability values (Table 1) is negative for all proteins, see Fig. 6d, left hand side axis. Note that these quantities were directly derived from the data plotted in Fig. 6c and are thus not independent. The energy difference is the energy necessary to overcome entropy, which favors free diffusion. The energy difference and the ratio of transition rates for all single data points are shown in Supplementary Fig. S4. Neither quantity depends on the molecular weight of the protein-mEGFP-construct, supporting the idea that the measured interaction is governed by the binding properties of each single protein instead of the protein size. For all proteins, the average ratio of the transition rates from free to bound, $r_{fb}$, and from bound to free, $r_{bf}$, is lower than 1, as shown in Fig. 6d, right hand side axis, suggesting that the transition from the bound state to the free state is more likely than vice versa. Thus, as expected from the average percentage of the bound pool, the proteins prefer the free state over the bound state. If the energy of the free state is influenced only by thermal energy, the energy of the bound state can be obtained in units of $k_B T$ using Eq. (7), see Supplementary Fig. S5. As expected, it is lowest for synapsin ($E_b$ = (2.5 ± 0.9) $k_B T$) and amphiphysin ($E_b$ = (2.8 ± 0.8) $k_B T$), the proteins that interact the most, and highest for calmodulin ($E_b$ = (4.2 ± 1.0) $k_B T$), the protein that interacts the least.

Our in vitro results for $D_{\text {bound}}$ are in agreement with literature values in living cells. For example, for calmodulin in HEK cells^11,13^ a range from 0.01 up to 10 μm^2^/s, depending on the measuring conditions and the type of fluorescent label used, i.e. EGFP^11^ or chemical dyes^13^, was reported. This wide range of diffusion coefficients found in HEK cells also reflects different experimental techniques employed, i.e. correlation spectroscopy versus single-molecule tracking. Additionally, calmodulin is found in different cellular compartments, and is involved in different transport mechanisms. Another example is complexin 1, for which a diffusion coefficient of 2 μm^2^/s, similar to our value, was measured in synapses of living *C. elegans*^12^. Additionally, we compared our in vitro data with our recent data measured by FRAP in living neurons^6^. To do so, we show the median diffusion coefficients of the bound component $D_{\text {bound}}$ together with median values derived from living neurons, see Fig. 6e. For the living neurons, we distinguished between values measured for the whole synapse (blue box plot) and for the vesicle cluster (magenta box plot). A Kruskal-Wallis test, followed by Tukey’s post-hoc test, confirmed the significant difference (*p*-value = 0.003, α-level = 0.05, *N* = 9, CALM is excluded here as it was not measured in living cells) between the mobility measured in vitro in the presence of the SV pattern and in the dense vesicle cluster in living neurons. This difference may be due to the geometry of our in vitro synapse model, which constitutes a 2D layer of vesicles that is in contrast to the dense 3D cluster of vesicles found in cells, which might further reduce protein mobility. When quantifying the vesicle density on our 2D patterns using STED microscopy (see Supplementary Fig. S6), we find about 15 vesicles per μm^2^, with a distance of about 177 nm between vesicle centers. In real synapses, the density in a plane taken through the vesicle cluster is higher, around 37 vesicles per μm^2^, and 93 nm between vesicle centers. This may at least to some extent explain, why we do not reproduce the values found deep in the vesicle cluster.

By contrast, when comparing our in vitro results, averaged over all proteins, to data from living cells for the whole synapse, they are very similar. Indeed, a Kruskal-Wallis test confirmed the non-significant difference, with a *p*-value of 0.08 (α-level = 0.05, *N* = 9, CALM is excluded here as it was not measured in living cells), between the mobility measured in the in vitro vesicle pattern and in the overall synapse in living neurons. We speculate that our in vitro results reproduce the situation in cell compartments, where the protein mobility is mostly Brownian, complemented by vesicle interaction. By contrast, if active transport or extreme confinement affected the protein mobility in the synaptic bouton, the diffusion coefficients of the proteins would differ substantially.

In summary, all eleven proteins, excluding EGFP, bind to the patterned SVs, and a clear decrease of mobility was measured in all cases (see Fig. 6a). We did not find a correlation between the average time spent bound (see Table 1) and the diffusion coefficients of the bound fraction, $D_{\text {bound}}$. However, for some of the proteins, we can speculate about their binding behavior as measured here and their biological role and function.

Amphiphysin (light blue in Figs. 5 and 6) is known to directly interact with the lipids in the SVs^27^, rather than binding protein partners as is the case for most other proteins. This aspect is reflected in our finding that amphiphysin is slowed down the most, spends the highest percentage of time bound to the SVs and consequently the binding energy $E_b$ is lowest. Proteins that require protein partners, and especially soluble co-factors, to bind to the vesicles, would in contrast be at a disadvantage, as our purified vesicles are mostly devoid of soluble co-factors. Calmodulin (red in Figs. 5 and 6) spends the least time bound to the vesicles and thus has the highest binding energy $E_b$ compared to the other proteins, which is to some extent expected, as this molecule is less strongly connected to presynaptic function than all others. Calmodulin is a general signaling component that is also involved in many vesicle-unrelated processes, such as postsynaptic dynamics^28^. Similarly, CALM (purple in Figs. 5 and 6) shows a low percentage of time it spends bound and therefore has a high binding energy $E_b$. It is known to bind the SV SNAREs directly. However, the interaction is dependent on the presence of phosphatidylinositol 4,5-bisphosphate (PIP$_2$), which is present in the SVs^29^, but not enriched as in the plasma membrane^30^. $\alpha$-synuclein (dark blue in Figs. 5 and 6) is known to bind SNAREs^31^ and may attach to them on the SVs, thus being slowed down resulting in a low value for $D_{\text {bound}}$ than the other proteins. We found synapsin to be the molecule that interacted most with the SVs. This molecule is capable of binding both SV proteins and SV lipids^32^ and its main function seems to be the tethering of vesicles to each other and to the actin cytoskeleton, being a main contributor to vesicle clustering^33^. It is therefore not surprising that it also displays a strong interaction in our assay.

## Conclusion

We established a minimalist in vitro model of the synaptic environment, designed by patterning SVs on glass coverslips. We employed FCS on fluorescently tagged synaptic proteins to quantify their mobility in the presence of SVs. Our approach is able to mimic the situation in the cell very closely and offers the additional advantage of high controllability and flexibility. For example, in future experiments, the design could be combined with microfluidic channels to add or wash-out reagents in a highly defined manner. The system further allows to quantitatively assess how the addition of kinases and phosphatases affects the kinetics of protein binding to SVs. We observed a clear decrease in mobility for proteins in the presence of the SVs, which we attribute to interaction events between the vesicles and the synaptic proteins. Overall, these data demonstrate that eleven different synaptic proteins, involved in different steps of SV exo- and endocytosis, can bind to SVs, which strongly reduces their mobility. Interestingly, the simple interaction to a monolayer of vesicles reduced the protein mobility to the values observed in living neurons to the level of the whole synapse^6^, thereby implying that SV interaction is a major controller of synaptic protein mobility. These experiments therefore provide an answer to the question of whether the SV cluster acts as a determinant of protein organization in the synapse, as described in the introduction, and provide further support to the hypothesis that the SV cluster forms a distinct phase in the synapse, which locally concentrates a plethora of proteins important for synaptic transmission^9^.

## Methods

### Glass coverslip functionalization

Glass coverslips (number 1, Thermo Scientific Technologies Inc., Wilmington, USA) were functionalized with neutravidin (Thermo Fisher Scientific, Waltham, MA, USA) to allow for the immobilization of SVs. The neutravidin was patterned on the glass coverslips using a photopattening system (PRIMO, Alvéole, Paris, France), mounted on top of an inverted microscope (Olympus IX83, Olympus Europa SE & CO. KG, Hamburg, Germany). The photopatterning system was calibrated in the beginning of each patterning day, following the manufacturer’s instructions. Glass coverslips were cleaned by rinsing them with isopropanol. After drying the coverslips with dry N_2_, they were treated with air plasma (ZEPTO, plasma cleaner, Diener Electronics GmbH, KG Ebhausen, Germany) for 3 minutes at 40 W. After the plasma treatment, a polydimethylsiloxane (PDMS) stencil creating a circular chamber (diameter 3.5 mm) designed to enclose the patterns, was applied to the surface, and 20 μL of PLL-g-PEG (0.1 mg/mL, PLL(20)-g[3.5]-PEG(2 kDa), SuSoS AG, Dübendorf, Switzerland) diluted in phosphate buffered saline (PBS) were added into the PDMS well and incubated for 1 h.

Figure 1a summarizes the main steps of the functionalization of the glass coverslips. The PLL-g-PEG coating provided the coverslips with anti-fouling properties, preventing unspecific protein adsorption. After rinsing three times with PBS, 8–10 μL of UV-sensitive photoinitiator (PLPP, Alvéole) were added into the PDMS well. To create the virtual mask with the pattern (in our case circular dots with 130 or 170 μm diameter), the open source software Inkscape (Inkscape Project, https://inkscape.org) was used. The pattern was loaded into the photopatterning software, Leonardo (Alvéole), and a 20 × objective (Olympus LUCPLFLN 20X, NA = 0.45) projected the UV light through the virtual mask. The PLPP, once activated by UV light, degraded the anti-fouling layer of PLL-g-PEG, leaving the exposed regions available for the attachment of neutravidin. A dose between 1800 and 2000 mJ/mm^2^ was used. After patterning, the PLPP was removed by washing three times with PBS, and neutravidin (concentration of 0.05 mg/mL) was added to the pattern in the PDMS well. We used fluorescently labeled neutavidin-FITC (concentration of 0.05 mg/mL) to check the quality of the patterned substrate. However, in the actual experiments, unlabeled neutravidin was used. The protein was incubated overnight at 4 °C, and, after washing off the remaining protein with PBS, the functionalized glass coverslips were ready to be used.

### Vesicle immobilization

Biotinylated mouse anti-synaptotagmin monoclonal antibodies (Synaptic Systems GmbH, Göttingen, Germany), were added to neutravidin-functionalized coverslips (concentration of 0.01 mg/mL) and incubated for 1 hour. SVs were purified from rat brain as previously described^5^ and incubated with anti-vGLUT1 single-domain antibodies (concentration of 0.05 mg/mL) labeled with STAR635P (Nanotag, Göttingen, Germany) for 1 hour. Subsequently, the pattern was washed with PBS 3 times and the labeled SVs were incubated on the pattern for 1 hour. The coverslips were then washed 3 times with PBS to remove unbound vesicles. A schematic representation of the resulting assembly is shown in Fig. 1b.

### STED imaging and vesicle density quantification

For quantification of the SV density, vesicles were immobilized on FITC-conjugated neutravidin patterns, as described above, and were stained with primary anti-synaptophysin and secondary anti-guinea pig antibodies conjugated to STAR635P. Imaging was performed using an Abberior easy3D STED microscope (Abberior GmbH, Göttingen, Germany) equipped with a UPlanSApo 100 ×, 1.4 NA oil immersion objective (Olympus) and an EMCCD iXon Ultra camera (Andor, Belfast, Northern Ireland, UK). A pulsed 640 nm laser was used for excitation, and an easy3D module 775 nm laser was used for depletion. Images were analyzed using a custom written Matlab script. In brief, the STED images were filtered using a bandpass filter to eliminate background noise and the spots above an empirically-defined threshold were identified. Their number was used to determine the vesicle densities, while their positions were used to measure the inter-vesicular distance. The example STED image presented in Supplementary Fig. S6 was processed by deconvolution, using in-built algorithms in Huygens Essential 4.4 (Scientific Volume Imaging, Hilversum, The Netherlands).

### Protein purification

The following proteins (with the respective mRNA reference sequence numbers indicated in parentheses) were simultaneously tagged with mEGFP for FCS measurements and ALFA-tag for affinity purification^21^: α-synuclein (NM_001009158.3), amphiphysin (NM_022217.1), clathrin assembly lymphoid myeloid leukemia (CALM) (AF_041374.1), calmodulin 1 (NM_031969.2), clathrin light chain B (NM_053835.1), complexin 1 (U35098.1), endophilin A1 (NM_053935.1), epsin (NM_057136.1), Rab3a (NM_013018.2), and Rab7a (NM_023950.3). HEK293 cells were transfected with the coding plasmids using Lipofectamine 2000 reagent (Invitrogen, Carlsbad, CA, USA) following the manufacturer’s recommendations. The proteins were expressed for approximately 24 hours and then purified using ALFA Selector PE-based chromatography. In brief, the cells were lysed with lysis buffer (1 % triton, 2 mM EDTA, protease inhibitor cocktail in PBS), the cell debris was pelleted by centrifugation and the supernatant was applied to the ALFA Selector PE resin. The ALFA-tagged proteins were allowed to bind to the resin for 1 h at 4 °C, while rotating, and all non-bound components were washed away twice with lysis buffer and once with ice-cold PBS. The bound proteins were then eluted with ALFA elution peptide. The presence of the protein of interest was confirmed by observing mEGFP fluorescence at every purification step and the resulting purified fractions were analyzed on a coomassie stained polyacrylamide gel (Supplementary Fig. S2). EGFP-Synapsin 1 and EGFP were expressed in Expi293F cells (Thermo Fisher Scientific) for three days following enhancement. Cells were harvested and lysed in buffer that contained 25 mM Tris-HCl (pH 7.4), 300 mM NaCl, 0.5 mM TCEP (buffer A), and protease inhibitor (cOmplete Protease Inhibitor Cocktail, EDTA-free, Roche, Penzberg, Germany). The lysates were centrifuged for 1h at 20,000×*g*, followed by a two-step purification. The first step was affinity purification on an Ni-NTA column (HisTrap HP, GE Healthcare Life Sciences, Freiburg, Germany) with binding at 20 mM, wash at 40 mM, and elution with 400 mM Imidazole in buffer A. The second step was size exclusion chromatography (Superdex 200 Increase 10/300, GE Healthcare) in 25 mM Tris-HCl (pH 7.4), 150 mM NaCl, 0.5 mM TCEP.

### Optical setup

The setup used was based on an inverted microscope (Olympus IX73, Olympus). The excitation light was provided by two pulsed diode lasers (Cobolt Samba-532 100 mW and Cobolt Calypso-491 25 mW, Cobolt AB, Solna, Sweden) inserted into a laser combiner box (C-Flex, Cobolt AB). After exiting the optical fiber, the laser light passed through a clean-up filter (HC Laser Clean-Up MaxLine 491/1.9 or HC Laser Clean-up MaxLine 532/2, AHF Analysentechnik AG, Tübingen, Germany). The laser beam was expanded by a factor of 10 in order to illuminate the full back aperture of the microscope objective. The laser intensity was attenuated with a neutral density filter (OD = 6, Qioptiq Photonics, Göttingen, Germany) before being deflected by a dichroic mirror (DualLine zt488/532rpc, AHF Analysentechnik AG) into the microscope. The laser beam was focused onto the sample using a 60× water immersion objective (UPlanApo, NA = 1.2, Olympus). The fluorescence light was then focused using an *f* = 200 mm lens onto the confocal pinhole (diameter 50 μm, Qioptiq Photonics). After the emission filter (Razor Edge Long Pass Filter 488 or RazorEdge LP Edge Filter 532, AHF Analysentechnik AG) the light was collimated using an *f* = 50 mm lens and directed to the avalanche photo diode ($\tau$-SPAD, Picoquant GmbH, Berlin, Germany). The $\tau$-SPADs were connected to a digital correlator card (ALV-7004 USB, ALV-Laser Vertriebsgesellschaft mbH, Langen, Germany). The digital correlator card was directly connected to the PC to store the data and then analyze them using Python (Python Software Foundation, https://www.python.org).

The same microscope was also used for epi-fluorescence microscopy. A mirror in the second deck of the microscope body enabled us to switch between the two microscope configurations. The excitation light came from a mercury arc lamp (X-Cite 120 PC Q, Excelitas Technologies, Waltham, USA) and was guided onto a fluorescence filter cube (filter sets available: DAPI, GFP, Cy3, TxRed and Cy5, all from AHF Analysentechnik AG). Images were acquired using a CCD-camera (Hamamatsu Orca R-2, Hamamatsu Photonics Deutschland GmbH, Herrsching am Ammersee, Germany) controlled by Micro-Manager^34^. To access different positions in the sample, an automated sample stage (Prior Scientific, Inc., Rockland, MA, USA) was used.

### Single-point FCS measurements

FCS measurements without the SVs were performed using about 250 μL of sample placed in an eight-well chamber slide (Nunc, Thermo Fisher Scientific). In the beginning of every measurement day, the setup was aligned and the observation volume was determined. The diameter of the observation volume, $w_0$, was calculated measuring the ACF of a well-characterized dye, Atto 488 (AttoTech GmbH, Siegen, Germany , *D* = (400 ± 10) μm^2^/s at 25 °C^35^) or Rhodamine 6G (Thermo Fisher, *D* = (414 ± 5) μm^2^/s at 25 °C^36^), at a concentration of 10 nM. The ACF, was fitted using a single-component model for diffusion^23^:

1 $$\begin{aligned} \text {ACF}(\tau ) = \frac{\gamma }{N} \frac{1}{1 + \frac{\tau }{\tau _D}} \frac{1}{\sqrt{1+ \frac{\tau }{\tau _D} (\frac{w_0}{z_0})^2}}, \end{aligned}$$

where $\tau$ is the correlation time, *N* is the average number of fluorescently labeled objects in the observation volume, $w_0$ and $z_0$ are the beam profile parameters of the observation volume, $\tau _D$ = $w_0/4 D$ is the diffusion time with *D* as the diffusion coefficient, and $\gamma$ is the illumination profile factor, which in our case is 0.35. The one-component ACF can also be rewritten as:

2 $$\begin{aligned} \text {ACF}(\tau ) = \frac{\gamma }{N}G(\tau ) = G(0) G(\tau ), \end{aligned}$$

where *G*(0) is the amplitude of the ACF at $\tau$ = 0, which contains information about the concentration of the sample. The measurements were performed at 22 °C. Typically, the diameter of the observation volume was $w_0$ = (310 ± 10) nm. Each protein was investigated using multiple samples with SV patterns. For each pattern, about 30 ACFs were recorded with an acquisition time of 30 s each.

For the measurements, where we detected two diffusing components, a two-component model^23^ was employed to describe the data:

3 $$\begin{aligned} \text {ACF}_2(\tau ) = \frac{\gamma }{N^2} \bigl ( N_1 G_1(\tau ) + N_2 G_2(\tau ) \bigr ), \end{aligned}$$

where $N_1$ and $N_2$ are the average numbers of fluorescently labeled objects of each species, $N = N_1 + N_2$ is the total number of diffusing objects and $G_1(\tau )$ and $G_2(\tau )$ correspond to the single species, see Eq. (2). The data were analyzed using a self-written fitting routine employing Python code. Measurements, where strong fluorescence peaks caused by aggregates affect the correlation curves, were excluded from the analysis. All correlation curves were fitted with a Levenberg-Marquardt nonlinear least-square routine.

### Evaluation of interaction energy

To describe the behavior of synaptic proteins in the presence of SVs, we assumed two states, a bound state *b* with an average energy $E_b$ and an unbound, or free, state *f* with an average energy $E_f$. We assumed that both states are always available to each copy of the proteins, i.e. no state is saturated or blocked by steric effects. The probability that a protein binds to an SV is $p_b$ and the probability that it freely diffuses is $p_f$. Since we allowed for enough time for the system to equilibrate before the measurement, we assumed a steady state distribution between the bound and the unbound state and free state. The partition function *Z* of the two states is

4 $$\begin{aligned} Z = \exp (-E_b/k_B T) + \exp (-E_f/k_B T), \end{aligned}$$

with the thermal energy of the system $k_B T$. The ratio of the probabilities to find the protein in the bound state $p_b$ or the free state $p_f$ is obtained via Boltzmann distributions

5 $$\begin{aligned} \frac{p_b}{p_f} = \frac{ \frac{1}{Z} \exp {(-E_b/k_B T)}}{\frac{1}{Z} \exp {(-E_f/k_B T})} = \exp ({(E_f -E_b)/k_B T}) . \end{aligned}$$

Thus, the energy difference between the two states can be expressed as

6 $$\begin{aligned} E_f - E_b = k_B T \ln (p_b/p_i) = k_B T \ln (p_b / (1-p_b)). \end{aligned}$$

In first approximation, for the free state, we assumed three degrees of freedom, $f=3$, per protein, i.e. only translational kinetic energy, and thus assigned the energy $E_f$ = $f/2 \cdot k_B T=3/2 \cdot k_B T$. Thus, for the energy of the bound state, we obtain

7 $$\begin{aligned} E_b = \frac{3}{2} k_BT - k_B T \ln (p_b / (1-p_b)). \end{aligned}$$

As we assume the system to be in equilibrium, the ratio of the transition rates $r_{fb}$ from the free state to the bound state and $r_{bf}$ from the bound state to the free state directly follows from Eq. (5):

8 $$\begin{aligned} \frac{r_{fb}}{r_{bf}} =\frac{p_b}{p_f}= \exp {\left( (E_f - E_b)/k_B T\right) }, \end{aligned}$$

and consequently,

9 $$\begin{aligned} r_{fb} = r_{bf} \cdot p_b/p_f . \end{aligned}$$

Thus, the transition from the free to the bound state is $p_b/p_f$ times slower than the reverse transition.

## Supplementary information

Supplementary Information.

## Data Availability

The datasets generated and/or analyzed during the current study are available from the corresponding author on reasonable request.

## Code availability

The codes generated for the analysis in the current study are available from the corresponding author on request.
