# Supplementary material for: A minimalist model to measure interactions between proteins and synaptic vesicles
Source: Sci Rep. 2020 Dec 3;10:21086. doi: 10.1038/s41598-020-77887-1 (PMC7713060; doi:10.1038/s41598-020-77887-1)
Supplement: Supplementary file 1 — Supplementary Information. [file 41598_2020_77887_MOESM1_ESM.pdf]

A minimalist model to measure interactions between proteins and synaptic vesicles

Eleonora Perego[1,+], Sofiia Reshetniak[2,+], Charlotta Lorenz[1], Christian Hoffmann[3], Dragomir Milovanović[3], Silvio Rizzoli[2,4], and Sarah Köster[1,4,*]

[1]Institute for X-Ray Physics, University of Göttingen, Göttingen, 37077, Germany
[2]Institute for Neuro- and Sensory Physiology, Center for Biostructural Imaging of Neurodegeneration, University Medical Center Göttingen, Göttingen, 37075, Germany
[4]Cluster of Excellence "Multiscale Bioimaging: from Molecular Machines to Networks of Excitable Cells" (MBExC), University of Göttingen, Göttingen, 37075, Germany
[3]Laboratory of Molecular Neuroscience, German Center for Neurodegenerative Diseases (DZNE), 10117 Berlin, Germany
[*]sarah.koester@phys.uni-goettingen.de
[+]these authors contributed equally to this work

Additional control measurements on patterned glass coverslips

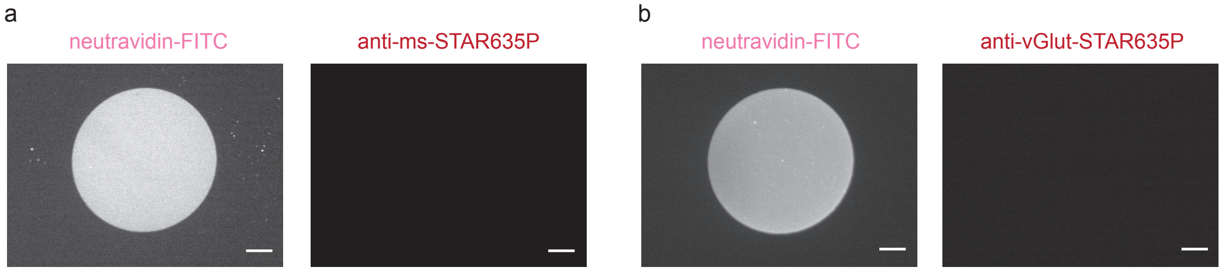

Figure S1. Negative controls on patterned glass coverslips without added SVs. a) After the neutravidin functionalization (neutravidin-FITC, left), the patterns were directly incubated with a secondary anti-mouse antibody labeled with STAR635P (right). b) After the neutravidin functionalization (neutravidin-FITC, left) and the incubation with biotinylated mouse anti-synaptotagmin antibody, a single-domain antibody against vGLUT1 labeled with STAR635P was added (without addition of SVs, right). Non-specific interactions are not observed in either case. The scale bars are 25 μm.

Coomassie stained polyacrylamide gel for the purified proteins

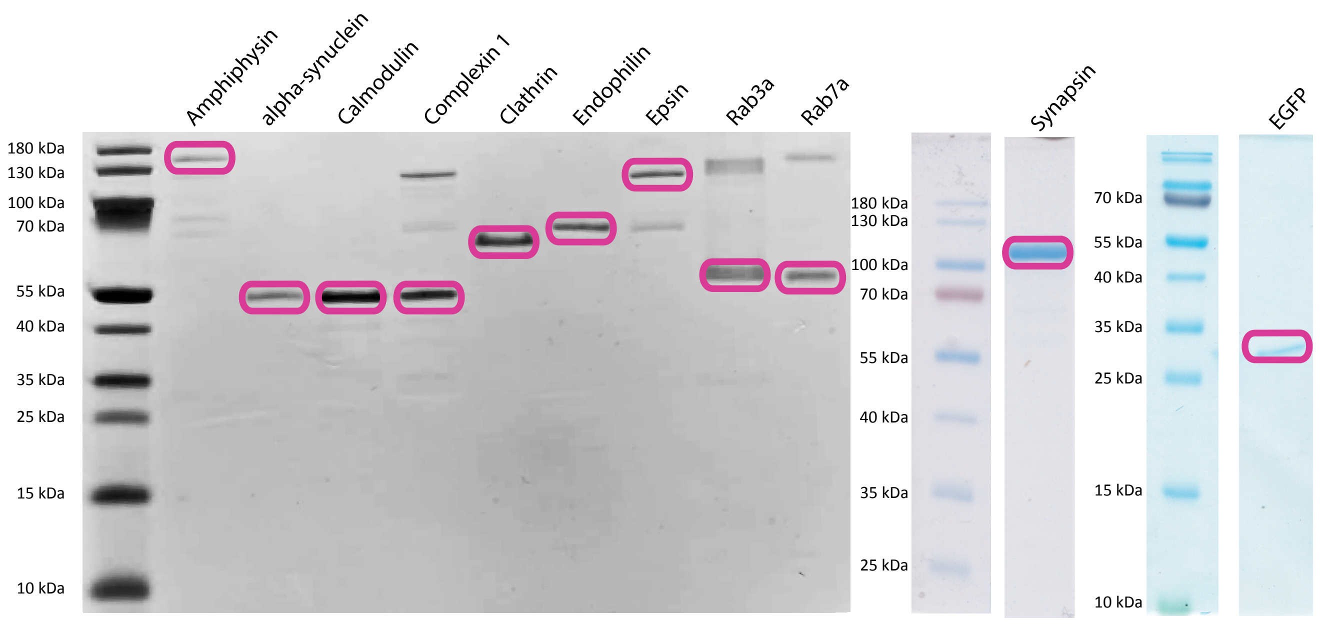

Figure S2. Coomassie stained polyacrylamide gel for all purified proteins tested in this study. Approximately 1 μg of protein was run on each lane, after protein purification (performed as shown in Fig. 3a). The behavior of the proteins is consistent with literature. Most proteins show one clear main band (indicated by the magenta boxes), found at the expected molecular weight. Synapsin and EGFP were run on separate polyacrylamide gels. Amphiphysin and epsin, and, to a lower extent, clathrin, run at higher molecular weights than their nominal values, as previously reported in the literature[1–3]. Some protein show secondary bands are that account for, on average, (11 $\pm$ 13) % of the total protein amounts. CALM was not analyzed in the same fashion, because this protein purifies to very low levels and is difficult to visualize by coomassie stained polyacrylamide gels. Additionally, CALM is known to display an unusual behavior during SDS-PAGE, separating into multiple bands of different molecular weights[4,5].

Comparison of the bulk diffusion coefficient and the free diffusion coefficient in the presence of SVs

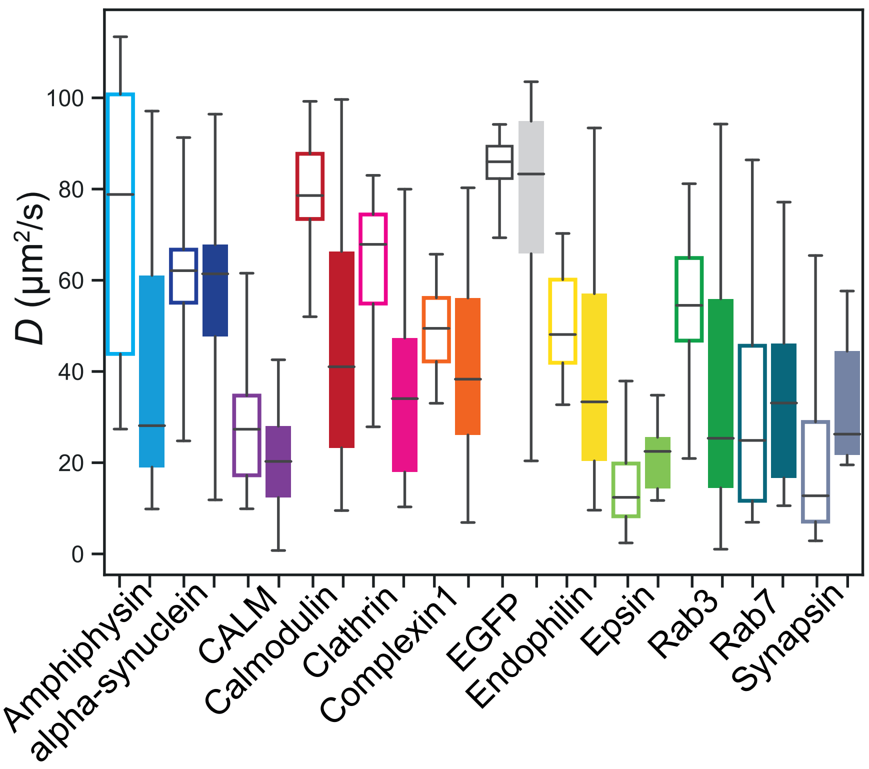

Figure S3. Diffusion coefficients of the proteins freely diffusing in bulk (D_{bulk}, empty boxes) and of the freely diffusing component in the SV patterns (D_{free}, filled boxes). The boxes extend from the lower quartile to upper quartile of the data, the middle lines in the box plots represent the median, the whiskers extend from the minimum to the maximum data point. The colors code represents the proteins as in the main text.

Results of the Mann-Whitney test to compare D_{bulk} and D_{bound}

Protein	p-value
Amphiphysin-mEGFP	2×10^{-12}
α-synuclein-mEGFP	5×10^{-20}
CALM-mEGFP	2×10^{-13}
Calmodulin-mEGFP	4×10^{-8}
Clathrin-mEGFP	2×10^{-13}
Complexin1-mEGFP	3×10^{-7}
Endophilin-mEGFP	2×10^{-15}
Epsin-mEGFP	2×10^{-8}
Rab3-mEGFP	1×10^{-12}
Rab7-mEGFP	1×10^{-9}
Synapsin-mEGFP	5×10^{-8}

Table S1. p-values for a Mann-Whitney statistical test, chosen because of the non-normality of the data, performed to compare the diffusion coefficients for proteins in bulk, D_{bulk}, and for the bound diffusing component in the SVs patterns, D_{bound}. All tested pairs show a p-value below the tolerance level of 0.05; thus we observe a significant difference for each protein. N varies between 20 and 90 data points depending on the protein.

Energy differences and transition rate ratios

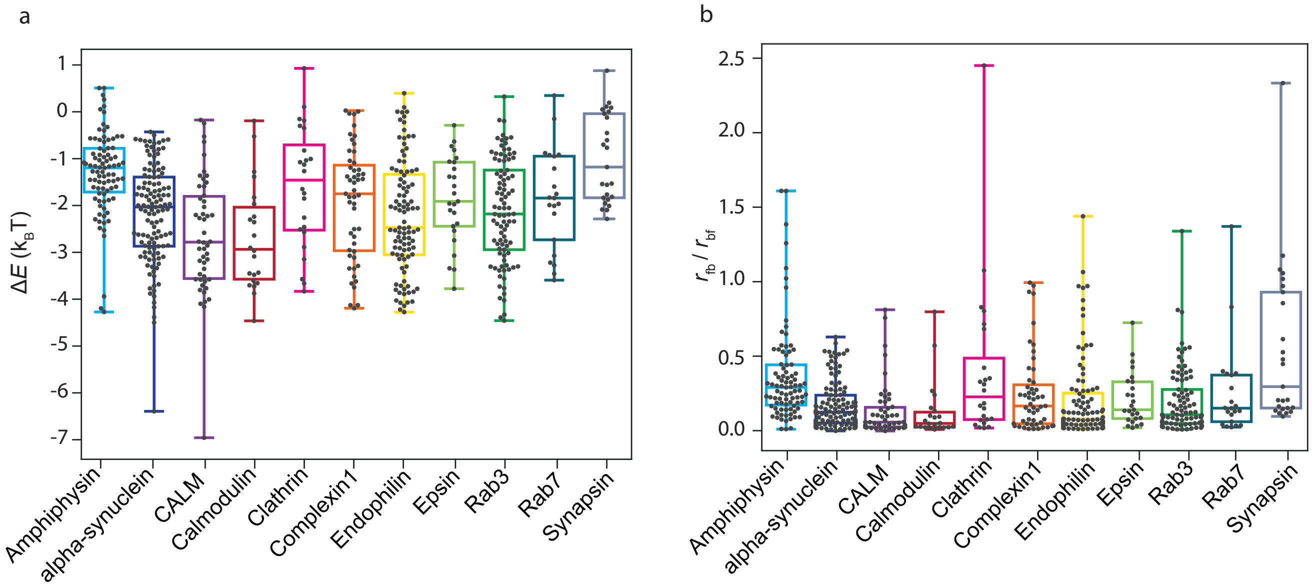

Figure S4. a) Energy difference between the free state and the bound state for all proteins. b) Ratio between the transition rate from the free state to the bound state and from the bound state to the free state. The boxes extend from the lower quartile to upper quartile of the data, the middle lines in the box plots represent the median, the whiskers extend from the minimum to the maximum data point.

Energies of the interaction state

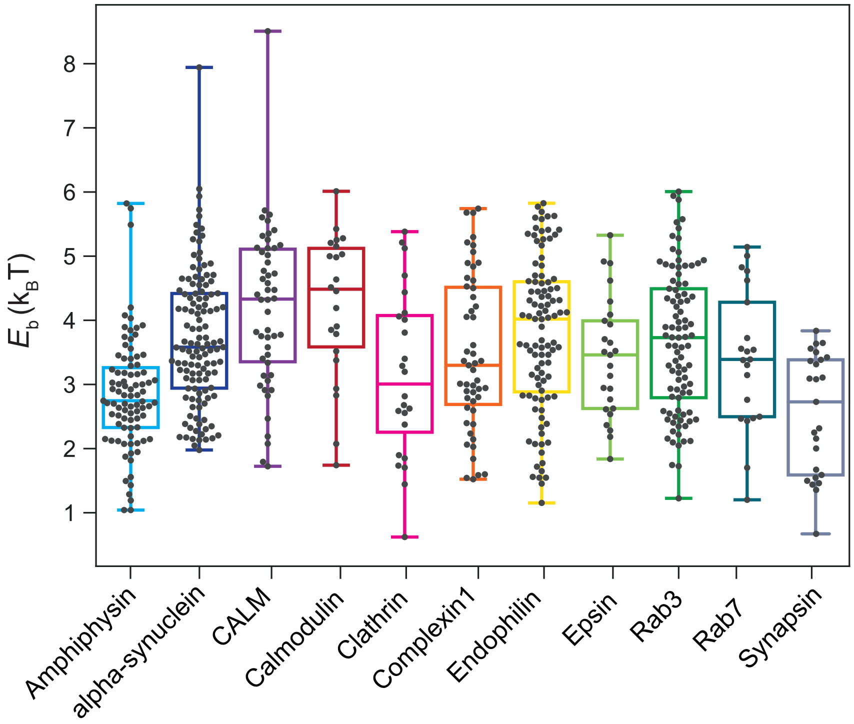

Figure S5. Estimated energy for the bound state for all measured proteins. The boxes extend from the lower quartile to upper quartile of the data, the middle lines in the box plots represent the median, the whiskers extend from the minimum to the maximum data point.

STED imaging of vesicle density

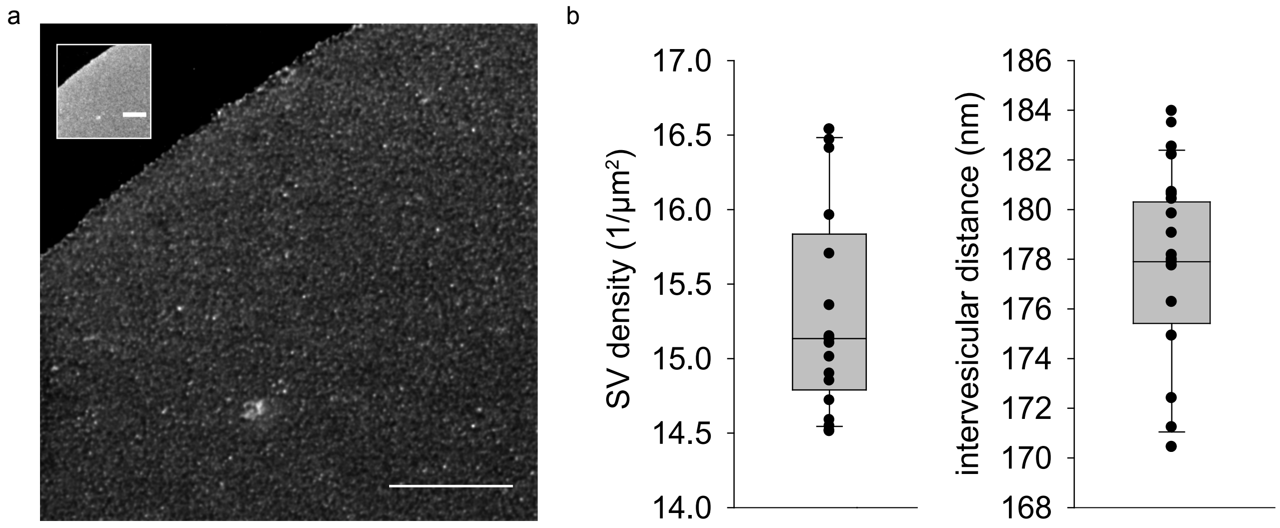

Figure S6. STED imaging of the patterned SVs to quantify the vesicle density. a) Representative STED image of labeled SVs attached to a neutravidin pattern. Inset: the respective confocal image of the underlying neutravidin-FITC pattern. Scale bars: 5 μm. b) Box plots of calculated vesicle densities (left) and distances between vesicle centers (right). 17 distinct pattern regions were analyzed, from one representative experiment (3 independent experiments in total. The middle line shows the median, the box edges indicate the 25th percentile, the error bars show the 75th percentile.).

References

1. Chen, H. *et al.* Epsin is an EH-domain-binding protein implicated in clathrin-mediated endocytosis. *Nature* **394**, 793–797, DOI: 10.1038/29555 (1998).
2. Laporte, S. A., Oakley, R. H., Holt, J. A., Barak, L. S. & Caron, M. G. The interaction of β-arrestin with the AP-2 adaptor is required for the clustering of β2-adrenergic receptor into clathrin-coated pits. *J. Biol. Chem.* **275**, 23120–23126, DOI: 10.1074/jbc.m002581200 (2000).
3. Slepnev, V. I., Ochoa, G.-C., Butler, M. H. & Camilli, P. D. Tandem arrangement of the clathrin and AP-2 binding domains in amphiphysin 1 and disruption of clathrin coat function by amphiphysin fragments comprising these sites. *J. Biol. Chem.* **275**, 17583–17589, DOI: 10.1074/jbc.m910430199 (2000).
4. Kim, J.-A. *et al.* Properties of GST-CALM expressed in e. coli. *Exp. Mol. Med.* **32**, 93–99, DOI: 10.1038/emm.2000.17 (2000).
5. Kim, J.-A. & Kim, H.-L. Cell-free expression and functional reconstitution of CALM in clathrin assembly. *Exp. Mol. Med.* **33**, 89–94, DOI: 10.1038/emm.2001.16 (2001).
